# Interfacing Seurat with the R tidy universe

**DOI:** 10.1093/bioinformatics/btab404

**Published:** 2021-05-24

**Authors:** Stefano Mangiola, Maria A Doyle, Anthony T Papenfuss

**Affiliations:** Bioinformatics Division, The Walter and Eliza Hall Institute, Parkville, VIC 3052, Australia; Department of Medical Biology, University of Melbourne, Melbourne, VIC 3010, Australia; Peter MacCallum Cancer Centre, Melbourne, VIC 3000, Australia; Sir Peter MacCallum Department of Oncology, University of Melbourne, Melbourne, VIC 3010, Australia; Bioinformatics Division, The Walter and Eliza Hall Institute, Parkville, VIC 3052, Australia; Department of Medical Biology, University of Melbourne, Melbourne, VIC 3010, Australia; Peter MacCallum Cancer Centre, Melbourne, VIC 3000, Australia; Sir Peter MacCallum Department of Oncology, University of Melbourne, Melbourne, VIC 3010, Australia; School of Mathematics and Statistics, University of Melbourne, Melbourne, VIC 3010, Australia

## Abstract

**Motivation:**

Seurat is one of the most popular software suites for the analysis of single-cell RNA sequencing data. Considering the popularity of the tidyverse ecosystem, which offers a large set of data display, query, manipulation, integration and visualization utilities, a great opportunity exists to interface the Seurat object with the tidyverse. This interface gives the large data science community of tidyverse users the possibility to operate with familiar grammar.

**Results:**

To provide Seurat with a tidyverse-oriented interface without compromising efficiency, we developed tidyseurat, a lightweight adapter to the tidyverse. Tidyseurat displays cell information as a tibble abstraction, allowing intuitively interfacing Seurat with dplyr, tidyr, ggplot2 and plotly packages powering efficient data manipulation, integration and visualization. Iterative analyses on data subsets are enabled by interfacing with the popular nest-map framework.

**Availability and implementation:**

The software is freely available at cran.r-project.org/web/packages/tidyseurat and github.com/stemangiola/tidyseurat.

**Supplementary information:**

Supplementary data are available at *Bioinformatics* online.

## 1 Introduction

**Fig. 1.**
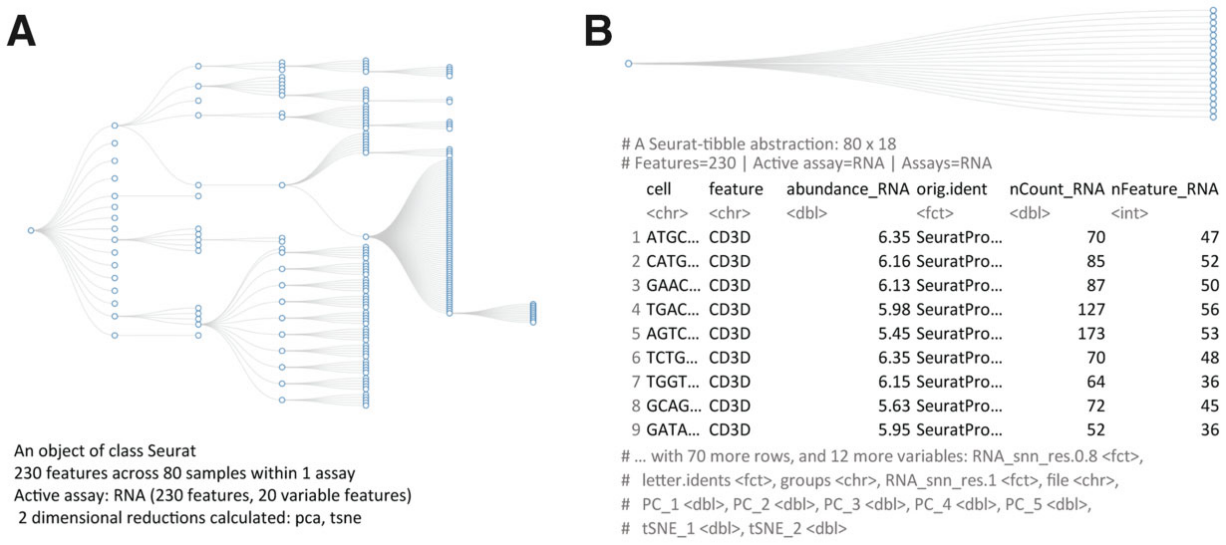
Comparison between the data structure (https://github.com/boxuancui/DataExplorer) (top; abstracted tibble for tidyseurat) and the information presented to the user (bottom) for Seurat (**A**) and tidyseurat (**B**; including transcript information). The dataset underlying these visualizations is a subset of a peripheral blood mononuclear cell fraction provided by 10× (10xgenomics.com)

Nucleotide sequencing at the single-cell resolution level has proven to be a disruptive technology that is revealing unprecedented insights into the role of heterogeneity and tissue microenvironment in disease (Keil *et al.*, 2018; Xiao *et al.*, 2019). Single-cell RNA sequencing data allows the robust characterization of tissue composition (Abdelaal *et al.*, 2019), the identification of cellular developmental trajectories (Chen *et al.*, 2019; Gojo *et al.*, 2020; Saelens *et al.*, 2019; Van den Berge *et al.*, 2020) and the characterization of cellular interaction patterns (Cabello-Aguilar *et al.*, 2020; Kumar *et al.*, 2018; Shao *et al.*, 2020). In recent years, the scientific community has produced many computational tools to analyze such data (Butler *et al.*, 2018; Lun* et al.*, 2016; McCarthy *et al.*, 2017). One of the most popular of these, Seurat (Butler *et al.*, 2018; Stuart *et al.*, 2019), stores raw and processed data in a highly optimized, hierarchical structure (Fig. 1A). This structure is displayed to the user as a summary of its content. The user can extract and interact with the information contained in such a structure with Seurat custom functions.

Machines and humans often have orthogonal needs when interacting with data. While machines prioritize memory and computation efficiency and favour data compression, humans prioritize low-dimensional data display and direct and intuitive data manipulation. Considering that low-dimensionality data representation often requires redundancy, balancing all priorities in a unique data container is challenging. Separating roles between the back-end data container and the front-end data representation is an elegant solution for ensuring transparency and efficiency. The scientific community has tackled this issue by offering visual and interactive representations of Seurat single-cell data containers. For example, Cerebro (Hillje *et al.*, 2020) is a Shiny-based standalone desktop application that enables the investigation and the inspection of pre-processed single-cell transcriptomics data without requiring bioinformatics experience. This application can import and export Seurat data containers.

Similarly, BioTuring (BioTuring INC) offers a visual web interface for facilitating data analysis by scientists without coding experience. NASQAR (Yousif* et al.*, 2020) (GitHub.com/nasqar) enables interactive analysis of a wide variety of genetic data, including single-cell RNA sequencing data from Seurat. Single Cell Viewer (SCV) (Wang *et al.*) is an R shiny application that offers users rich visualization and exploratory data analysis options for single-cell datasets, including Seurat. Although these tools allow an intuitive data representation and analysis, they are not fully programmable and pose a challenge for reproducibility. Moreover, they are generally less expandable than code-based tools, posing a challenge for the scientific community’s contribution. For example, as it is in the case of R data analysis repositories such as CRAN (Ripley, 2001) and Bioconductor (Huber *et al.*, 2015).

Recently, the data science community has made efforts towards the representation and manipulation of data using the concept of data tidiness (Wickham *et al.*, 2019). This paradigm allows the organization of information as a two-dimensional, highly flexible table (referred to as tibble, a type of data frame), with variables oriented in columns and observations oriented in rows. This new standard has become extremely popular across fields of data science. The application of tidiness principles would be compelling if applied to single-cell transcriptomic data. A tidy data structure would capture how biological data measurements relate to experimental design and metadata (e.g. technical and clinical properties of transcripts, cells and biological replicates). The shift from a compressed and hierarchical to a tabular data representation of cell- (by default) and (optionally) transcript-related information has two advantages. More extensive data display improves scientific awereness, and its tabular representation enables interfacing with a large ecosystem of tidy-oriented APIs for data manipulation and visualization. This interface facilitates data analysis and reproducibility for researchers across a broad spectrum of computational literacy. For example, tidyseurat allows to display, plot, modify, join, delete, filter, subsample, nest and summarize information of a Seurat object without leaving the tidyverse syntax. These functionalities offer a compelling synergy with the tidy counterpart for Bioconductor’s SingleCellExperiment objects (Amezquita *et al.*, 2020), tidySingleCellExperiment (available at bioconductor.org), moving towards a unified interface for single-cell data containers. As for comparison, although the indirect interface between Seurat objects and the tidyverse is possible, it requires intermediate steps to extract information that can be passed to downstream APIs. For example, building a custom plot that integrates reduced dimensions with cell-wise annotations (e.g. library size) requires integrating multiple data frames with custom routines (e.g. direct querying for metadata and embeddings for reduced dimensions).

Here, we present tidyseurat, an adapter that interfaces Seurat, a popular single-cell RNA sequencing data analysis tool, with tidyverse, a popular R data analysis framework. Although the data container is Seurat’s, tidyseurat displays a tibble abstraction that contains cell-wise information (Fig. 1B). Tidyseurat includes adapters to most methods included in dplyr (Wickham *et al.*, 2019), a powerful grammar of data manipulation; tidyr an extensive collection of methods for data reshaping and grouping; ggplot2, the most popular R visualization tool; and plotly, a powerful tool for interactive visualizations. As a result, the user can perform efficient analyses using Seurat (and Seurat-compatible software) while visualizing, manipulating, integrating and grouping the data using tidyverse (-compatible for plotly) software. This package is aimed at analysts of single-cell data who favor the use of tidyverse and Seurat. Tidyseurat is part of a larger ecosystem called tidytranscriptomics that aims to bridge the transcriptomics and tidy universes (github.com/stemangiola/tidytranscriptomics).

## 2 Materials and methods

### 2.1 Data user interface

Tidyseurat abstracts the complexity of the data container and provides a friendlier interface for the user. Tidyseurat implements an improved data display method (replacing the Seurat ‘show’ method), mapping the cell-wise information into a user-friendly table. By default, cell-wise information is displayed to the user (e.g. cell-cycle phase, cluster and cell-type annotation), leaving the transcript information available upon request using the ‘join_features’ function. This function adds transcript identifiers, transcript abundance and transcript-wise information (e.g. gene length, genomic coordinates and functional annotation) as additional columns. Cell-wise information is prioritized over transcript-wise information on the rationale that it is more often directly queried.

**Fig. 2.**
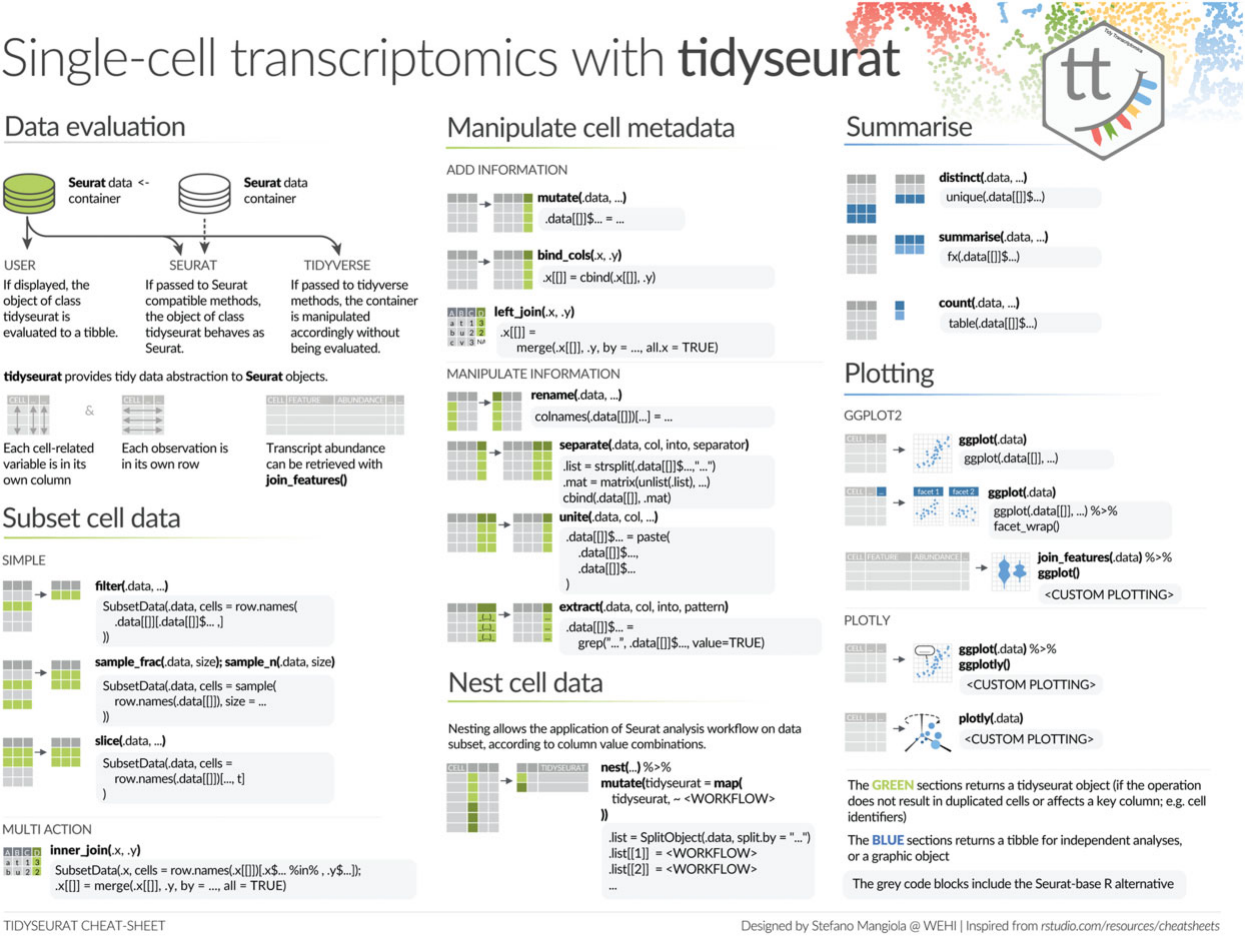
A cheat sheet of the tidyverse functionalities that tidyseurat enables for Seurat objects. This cheat sheet provides examples of the alternative tidyverse and Seurat syntax. The green colour scheme includes procedures that output a tidyseurat, if: (i) do not lead cell duplication; and (ii) key columns (e.g. cell identifier) are not excluded, modified, nor renamed (e.g. through a select, mutate and rename commands). In this case, a table (rather than an abstraction) is returned for independent analysis and visualization. The blue colour scheme includes procedures that return tibble tables for independent analyses and plotting. The grey-shaded boxes include the alternative code utilizing Seurat and base-R.

The tidyseurat tibble abstraction includes two types of columns, columns that can be interacted with and modified and columns that are view only. The editable columns are part of the cell metadata, while the view-only columns include data-derived variables, such as reduced dimensions (e.g. principal component and UMAP dimensions). The default integration of all cell-wise information, including reduced dimensions, in one tibble representation, facilitates data visualization, filtering and manipulation. To allow the manipulation and plotting of the data using the tidyverse ecosystem, the dplyr, tidyr, ggplot2 and the tidyverse-compatible plotly routines have been adapted to work seamlessly with the back-end Seurat data structure, allowing the user to operate as if it was a standard tibble. This abstraction strategy allows the data to appear as a tibble for end-users and the tidyverse (Table 1) but appear as a Seurat container for any other algorithm, thus preserving full backward compatibility (Fig. 2).

**Table 1.** Example of a tibble abstraction of a Seurat table

# A Seurat-tibble abstraction: 8033 × 11						
# Features = 1000 | Active assay=SCT | Assays=RNA, SCT						
Cell	Total count	Total transcripts	PC1	UMAP1	Cluster	Cell type
cell_1	10 456	450	−1.23	−3.47	1	T cell
cell_2	2088	400	0.98	−1.59	2	B cell
cell_3	11 309	699	5.55	1.26	5	Monocyte
cell_4	8791	423	−5.42	−4.42	1	Monocyte

*Note*: Pre-existing cell-wise annotation and newly calculated information are all coexisting in a unique table.

### 2.2 API user interface

The seamless integration with the tidyverse is obtained through adapters for most methods in the packages dplyr, tidyr, ggplot2, as well as plotly (Fig. 2). These methods belong to three groups based on the action that they perform on the back-end Seurat container. Methods such as ‘mutate’, ‘left_join’, ‘separate’, ‘unite’, ‘extract’ and ‘select’ manipulate or subset the information present in the cell-wise metadata. Methods such as ‘slice’, ‘filter’, ‘sample_n’, ‘sample_frac’, ‘inner_join’ and ‘right_join’ subset cells. Methods such as ‘bind_rows’ join two or more datasets. All these methods return Seurat objects (abstracted as tibbles) if those procedures do not lead to cell duplication and if key columns (e.g. cell identifier) are present. Otherwise, these methods return a tibble for independent analyses. Another group of functions such as ‘summarise’, ‘count’, ‘distinct’, ‘join_features’ and ‘pull’ return a tibble or an array for independent analyses. Tidyverse-compatible visualization methods include ggplot and plotly. The tidyseurat data abstraction allows the use of the nest-map tidyverse framework. Briefly, nesting divides tables into subsets according to any reference column, while the map function allows applying operations across subsets iteratively.

## 3 Algorithm and implementation

**Fig. 3.**
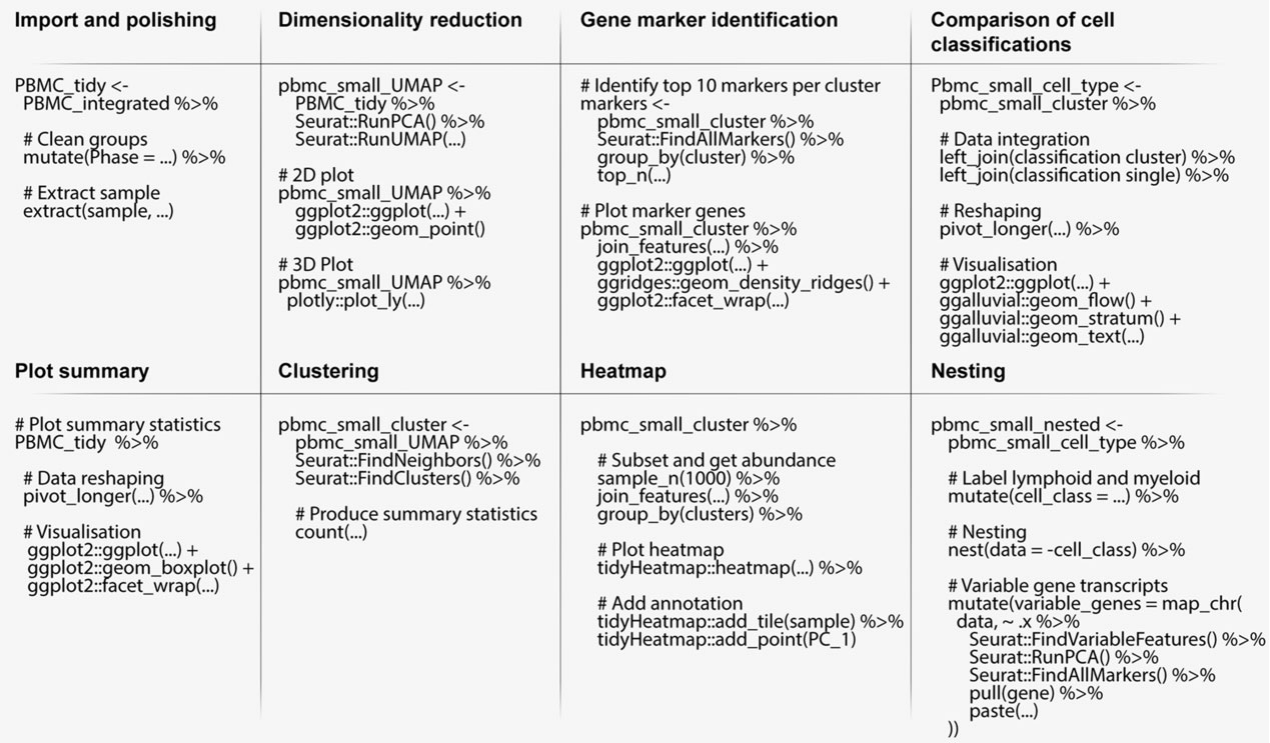
Pseudo-code representing procedures for the analysis of single-cell RNA sequencing data integrating Seurat and tidyverse functions through tidyseurat. For functions that are not part of tidyseurat nor base R, package prefixes were added

**Fig. 4.**
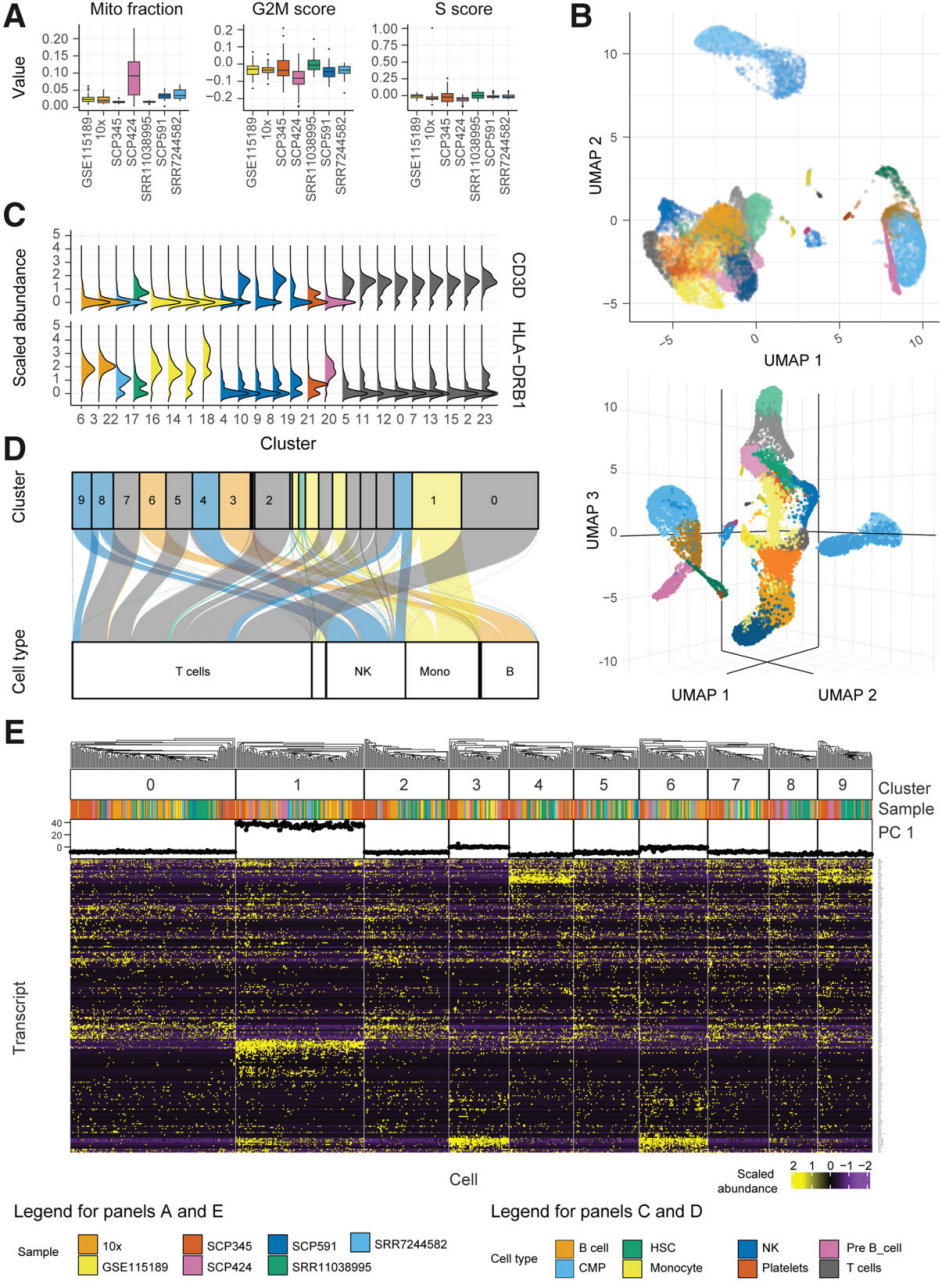
Tidyverse-compatible libraries offer powerful, flexible and extensible tools to visualize single-cell RNA sequencing data. Natively interfacing with such tools expands the possibilities for the user to learn from the data. Graphical results of the example workflow, integrating Seurat and tidyverse with tidyseurat. (**A**) Sample-wise distribution of biological indicators, including the proportion of mitochondrial transcripts and cell-cycle phase scores. For optimum visualization, a 20% subsampling was performed on the cell set. (**B**) Cells mapped in two- and three-dimensional UMAP space. The default integration of reduced dimensions and other cell-wise information in a tibble abstraction facilitates such visualization. (**C**) Distribution of transcript abundance for two marker genes, identified for each cluster identified by unsupervised estimation. Cells mapped in two-dimensional Uniform Manifold Approximation and Projection (UMAP) space. (**D**) Mapping of cells between the cell- or cluster-wise methods for cell-type classification. Only large clusters are labelled here. The colour scheme refers to cell types classified according to clusters. The bottom containers refer to the classification based on single cells. (**E**) Heatmap of the marker genes for cell clusters, produced with tidyHeatmap, annotated with data source and the first principal component. Only the ten largest clusters are displayed. The integrated visualization of transcript abundance, cell annotation and reduced dimensions is facilitated by the ‘join_features’ functionality and by the default complete integration of cell-wise information (including reduced dimensions) in the tibble abstraction

To demonstrate the use of tidyseurat, we provide as an example an integrated analysis of peripheral blood mononuclear cells from public sources. We show the main steps of a typical workflow, along with code examples (Fig. 3) and tidyverse-compatible visualizations (Fig. 4). We show how data manipulation and filtering can reduce coding lines and temporary variables compared to Seurat alone.

### 3.1 Data import, polishing and exploration

The single-cell RNA sequencing data used in this study consists of seven datasets of peripheral blood mononuclear cells, including GSE115189 (Freytag *et al.*, 2018), SRR11038995 (Cai *et al.*, 2020), SCP345 (singlecell.broadinstitute.org), SCP424 (Ding *et al*., 2020), SCP591 (Karagiannis *et al.*, 2020) and 10×-derived 6K and 8K datasets (support.10xgenomics.com/). In total, they include 50 706 cells. Data exploration is a crucial phase of any analysis workflow. It includes data curation, visualization and summary statistics, combined with dimensionality reduction and data scaling. Tidyverse commands allow intuitive manipulation and polishing of cell-wise annotations (cell-cycle phase and sample name) from the data abstraction (Fig. 3, import and polishing; Supplementary code chunk 2). Cell properties included in the resulting table (e.g. proportion of mitochondrial transcripts and cell-cycle phase; Fig. 4A) can be visualized in a faceted and integrated fashion using standard tidyverse tools (Fig. 3, Plot summary). This visualization facilitates quality control, helping identify potential low-quality samples such as SCP424 (Fig. 4A).

### 3.2 Dimensionality reduction

Dimensionality reduction allows visualizing cell heterogeneity in a low dimensional space (Fig. 3, Dimensionality reduction). Methods such as uniform manifold approximation and projection (UMAP) (McInnes *et al.*, 2018) define local cell similarities while preserving global distances. Seurat and tidyverse methods can be seamlessly integrated through tidyseurat to calculate and visualize UMAP dimensions (Fig. 3, Dimensionality reduction). The reduced dimensions are displayed as additional columns (view only) of the tidyseurat table. The use of tidyverse (Wickham *et al.*, 2019) for visualization allows great customization of two-dimensional plots (Fig. 4B). The advantage of tidyseurat here is the presence of cell annotation and reduced dimensions in the same data frame, which can be used for arbitrarily complex annotated visualizations (Supplementary code chunk 3). Tidyseurat enables the three-dimensional cell visualization with plotly (Sievert, 2020) (Fig. 4B). Displaying a third reduced dimension confers better awareness of cell heterogeneity and clustering. Dimensionality reduction shows three main cell clusters and a minor intermediate cell cluster (Fig. 4B, top). The larger cluster (bottom-left) includes 69% of all cells (https://github.com/stemangiola/tidygate). The display of the third UMAP dimension in an interactive environment gives an additional perspective on cell heterogeneity compared to only calculating and visualizing the first two (Fig. 4B, bottom).

### 3.3 Clustering and marker genes identification

Unsupervised clustering is essential to define self-similar groups of cells quantitatively. Similar to previous procedures, Seurat and tidyverse commands can be concatenated through inference and visualization (Fig. 3, Clustering; Supplementary code chunk 4). The newly calculated cluster identities will be displayed as additional columns in the tidyseurat table. Marker genes can be identified using cell clustering information (Fig. 3, Gene marker identification). Gene marker identification can be performed with Seurat, and transcript abundance distribution can be visualized for selected marker genes in a faceted and integrated manner using tidyverse (Fig. 4C). The advantage of tidyseurat here is the ease of integrating the transcript and cell information in the same data frame (through ‘join_features’) for joint manipulation, filtering and visualization (Supplementary code chunk 5). Heatmaps can visualize cell-wise transcript abundance for marker genes. While it is possible to use the Seurat integrated heatmap function (DoHeatmap), the tidyverse-style heatmap method (Mangiola and Papenfuss, 2020), tidyHeatmap, allows for more flexibility. For example, cell-wise data (e.g. principal components) can be used as annotations, choosing among four representations (e.g. tile, point, line and bar; Fig. 4E). The integration of diverse information facilitates quality check and curation. Shared-nearest-neighbour (SNN) method (Ertöz *et al.*, 2003) for unsupervised clustering identified 24 cell clusters with default settings. The largest cluster includes 17% of cells. The largest supercluster includes 69% of all cells and encompasses 18 clusters.

### 3.4 Cell type inference

While the classification of cell clusters in cell-type categories can be performed manually by analyzing marker genes, the automatic cell or cluster classification can represent a critical first step in the process. Several methods are publicly available (Alquicira-Hernandez *et al.*, 2019; Jaitin *et al.*, 2014; Kim *et al.*, 2019; Nagendran *et al.*, 2018; Tan and Cahan, 2019), including label transfer from publicly available annotated datasets (MapQuery functionality, satijalab.org). SingleR (Aran *et al.*, 2019) is a popular tool to classify both clusters and single cells using transcriptional references. While using cluster identity to drive the cell-type classification can benefit from data aggregation and improve the overall robustness of the inference, it relies on the goodness of clustering and the assumption that cells within the same cluster are of the same type. On the contrary, single-cell classification avoids biases due to clustering but introduces challenges relative to the absence of data hierarchy. Using tidyseurat, the consistency between these two methods can be visually and quantitatively checked (Fig. 3, comparison of cell classification). The tidyverse-style alluvial visualization is ideal for communicating the differences in classification with or without cluster information and integrates with the tidy data structure (Bojanowski and Edwards, 2016; Brunson, 2020; Kennedy and Sankey, 1898) (Fig. 4D). Using the Human Protein Atlas reference (Uhlén *et al.*, 2015), eight cell types were identified in total (including platelets, T, B, pre-B, natural killer, monocyte, myeloid progenitor and hematopoietic stem cells). For both classification approaches (cluster- or cell-wise), the most abundant cell type was T cells, including on average 51% of all cells. In total, 9.4% of cells were classified differently between the two methods (Fig. 4D).

### 3.5 Nesting

Subsetting the data according to sample, cell identity or batch is a common step of a standard analysis workflow. For example, grouping cells according to major cell subtypes (e.g. lymphocytes, myeloid and stromal cells) is helpful to improve the resolution of the analyses. Also, performing independent analyses across biological replicates can be helpful to assure that data integration is not creating artefacts. Similarly, balanced subsampling across biological replicates might be needed for an unbiased visualization of reduced dimensions. This grouping can be obtained by manually splitting the data into subsets according to a variable and iteratively applying procedures to each subset. Tidyverse gives a more powerful and intuitive framework to perform such operations on tibbles. According to any combination of variables, the function nest allows for nesting data subsets into a table column. The function map allows iterating procedures across such subsets without leaving the clear and explicit tibble format. An example is shown (Table 2; Fig. 3, Nesting) where (i) cell types are grouped in lymphoid and myeloid, and (ii) variable gene transcripts are independently identified for each of the two cell populations without the need to create any temporary variable.

**Table 2.** Example of a nested tidyseurat table, with gene markers calculated internally for each major immune cell type

# A tibble 2 × 3		
Cell class	Data	Top markers
lymphoid	<tidyseurat>	RPL34, RPS27, RPL32, RPS3A, RPL21, RPL31
myeloid	<tidyseurat>	S100A8, S100A9, S100A12, VCAN, CYP1B1, CD14

*Note*: This nesting is obtained with the nest-map combination from tidyverse.

### 3.6 The difference in coding style to Seurat

**Fig. 5.**
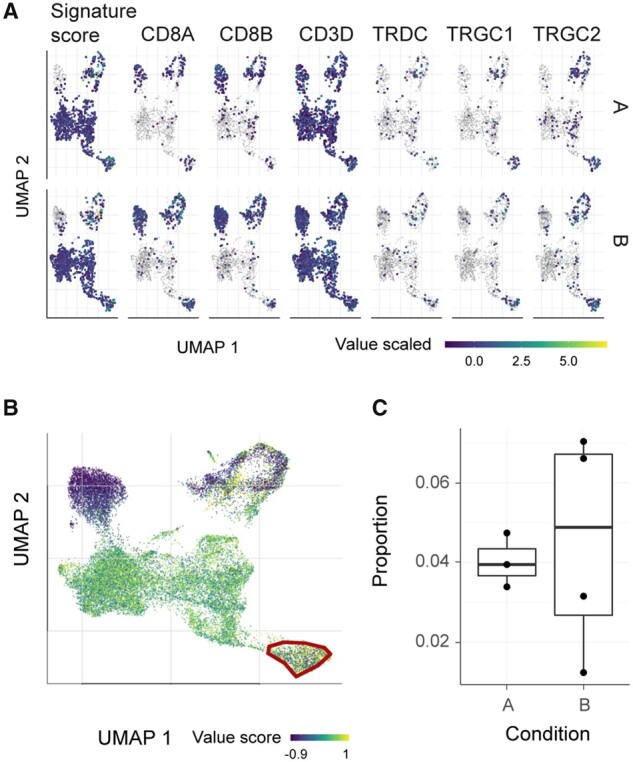
Presence of gamma delta T cells among lymphocytes, part of the case study for comparing Seurat with tidyseurat. (**A**) Integrative UMAP plot including both the signature score and the genes within the signature. Plots are faceted horizontally for biological condition (artifactual). (**B**) Interactive gating of high scoring cells for the gamma delta T cell signature (Pizzolato *et al.*, 2019), using tidygate (github.com/stemangiola/tidygate). (**C**) Distribution of the proportion of gamma delta T cells across patients from conditions A and B

Tidyseurat expands the tools available for interacting with Seurat data containers, especially for analyses not included in the standard Seurat framework. We present a case study where a single-cell RNA sequencing dataset (see Section 3) is analyzed for the presence of gamma delta T cells using a multiple-gene score (Pizzolato *et al.*, 2019). For this case study, samples were assigned to two groups (A or B). The study consists of five steps: (i) the score for the transcriptomic signature of gamma delta T cell is calculated; (ii) a balanced subsampling of cell across samples is taken for visualization; (iii) cells are visualized in UMAP reduced dimensions, faceting for both for their score and transcript abundance (Fig. 5A); (iv) cells with high gamma delta T cell score are manually gated (github.com/stemangiola/tidygate; Fig. 5B) and (v) proportions of gamma delta across samples are calculated, and sample groups are compared using a boxplot (Fig. 5C). Table 3 shows that tidyseurat allows a 1.4-fold reduction of lines of code (24 for Seurat versus 17 for tidyseurat) and a 9-fold reduction of variable assignment (9 for Seurat versus 1 for tidyseurat). Lines are evaluated as expressing one command [e.g. function(data) %>%].

**Table 3.** Case study for the detection of gamma delta T cells among lymphoid cells

Step	Seurat	tidyseurat
**Create and visualize signature in UMAP dimension**		
**Score signature**	signature_score_1 = seurat_obj[c(“CD3D”, “TRDC”, “TRGC1”, “TRGC2”),] %>% Seurat::GetAssayData(assay=“SCT”, slot=“data”) %>% colSums() %>% scales::rescale(to=c(0,1)) signature_score_2 = seurat_obj[c(“CD8A”, “CD8B”),] %>% Seurat::GetAssayData(assay=“SCT”, slot=“data”) %>% colSums() %>% scales::rescale(to=c(0,1)) seurat_obj$signature_score = signature_score_1 - signature_score_2	seurat_obj_sig = seurat_obj %>% join_features( features = c(“CD3D”, “TRDC”, “TRGC1”, “TRGC2”, “CD8A”, “CD8B”), shape = “wide”, assay = “SCT” ) %>% mutate(signature_score = scales::rescale(CD3D + TRDC + TRGC1 + TRGC2, to=c(0,1)) - scales::rescale(CD8A + CD8B, to=c(0,1)) )
**Subsample**	splits = colnames(seurat_obj) %>% split(seurat_obj$sample) min_size = splits %>% sapply(length) %>% min() cell_subset = splits %>% lapply(function(x) sample(x, min_size)) %>% unlist() seurat_obj = seurat_obj[, cell_subset]	seurat_obj_sig %>% add_count(sample, name = “tot_cells”) %>% mutate(min_cells = min(tot_cells)) %>% group_by(sample) %>% sample_n(min_cells) %>%
**Plot**	Seurat::FeaturePlot( seurat_obj, features = c(“signature_score”, “CD3D”, “TRDC”, “TRGC1”, “TRGC2”, “CD8A”, “CD8B”), split.by = “type”, min.cutoff = 0.1 )	pivot_longer(cols=c(“CD3D”, “TRDC”, “TRGC1”, “TRGC2”, “CD8A”, “CD8B”, “signature_score”)) %>% group_by(name) %>% mutate(value = scale(value)) %>% ggplot(aes(UMAP_1, UMAP_2, color=value)) + geom_point() + facet_grid(type∼name)
**Gate cells and visualize cell proportions biological conditions**		
**Gate cells**	p = Seurat::FeaturePlot(seurat_obj, features = “signature_score”) seurat_obj$within_gate = colnames(seurat_obj) %in% CellSelector(plot = p) seurat_obj[[]] %>% # Pass object to plot	seurat_obj_sig %>% mutate(gamma_delta = tidygate::gate_chr( UMAP_1, UMAP_2, .color = signature_score )) %>%
**Proportion (common)**	add_count(sample, name = “tot_cells”) %>% count(sample, type, tot_cells, within_gate) %>% mutate(frac = n/tot_cells) %>% filter(within_gate == T) %>%	
**Plot (common)**	ggplot(aes(type, frac)) + geom_boxplot() + geom_point()	

*Note*: Both Seurat and tidyseurat style coding is shown.

## 4 Discussion

Seurat is the most popular single-cell RNA sequencing data analysis workflow. It includes user-friendly methods for data analysis and visualization. Data query, manipulation and visualization require Seurat-specific functions. The R data-science community has settled on a robust, consistent and modular data representation, referred to as tidy. Tidyseurat exposes the data from the complex hierarchical structure of a Seurat object in the form of a tidy table. As a result, most of the data is readily visible to the user, who can leverage the large computational and visualization tidy ecosystem. Considering that tidyverse syntax and vocabulary (e.g. dplyr and tidyr) is becoming common knowledge, tidyseurat diminishes the domain-specific bioinformatic knowledge required to operate with Seurat objects. Most importantly, the full compatibility with the Seurat ecosystem is not compromised. By default, Seurat provides a wide range of custom methods for data plotting. The customizability of these methods is necessarily limited and achieved through setting command parameters. The tidyverse includes an increasing number of connected modules for data visualization that the tidy data representation can leverage, complementing or replacing custom methods. The amount of information and heterogeneity within single-cell RNA sequencing data often requires data subsetting and reanalysis. For example, highly diverse broad cell populations such as lymphoid and myeloid are often subset and analyzed independently to decrease the inference complexity and increasing resolution. While it is commonly required to manually subset the Seurat object, perform iterative analysis for each subset, and reintegrate the objects (if necessary), the tidy abstraction enables performing this more efficiently using the nest-map paradigm. This elegant and powerful paradigm allows self-contained and robust iterative analysis of data subsets. Tidyseurat is a standalone adapter that improves analysis reproducibility and scientific awareness, in a user-friendly way, without changing the user’s familiar Seurat analysis workflow. As the display and manipulation are centred on cell-wise information by default, the use of tidyseurat does not add any perceptible overhead. This approach is compelling in moving towards a unified interface for single-cell data containers, with a tidy container for SingleCellExperiment objects, tidySingleCellExperiment, now also available. We anticipate that this data abstraction will also be the pillar of more extensive analysis-infrastructures based on the tidy paradigm, such as has happened for bulk RNA sequencing data (Mangiola *et al.*, 2021). In summary, tidyseurat offers three main advantages: (i) it allows tidyverse users to operate on Seurat objects with a familiar grammar and paradigm; (ii) it streamlines the coding, resulting in a smaller number of lines and fewer temporary variables compared with the use of Seurat only and (iii) it provides a consistent user interface shared among other tidy-oriented tools for single-cell and bulk transcriptomics analyses (e.g. tidySingleCellExperiment and tidySummarizedExperiment at github stemangiola/tidySingleCellExperiment and stemangiola/tidySummarizedExperiment). The package tidyseurat offers extensive documentation through methods description, vignettes [accessible through typing browseVignettes(‘tidyseurat’)], and through workshop material (e.g. rpharma2020_tidytranscriptomics, ABACBS2020_tidytranscriptomics at github/stemangiola).

## Supplementary Material

btab404_Supplementary_Data
