# Supplementary material for: Interfacing Seurat with the R tidy universe
Source: Bioinformatics. 2021 May 24;37(22):4100–7. doi: 10.1093/bioinformatics/btab404 (PMC9502154; doi:10.1093/bioinformatics/btab404)
Supplement: btab404_Supplementary_Data [file btab404_supplementary_data.docx]

Supplementary material

## Supplementary code chunk 1

| library(Seurat)  library(SeuratObject)  library(tidyseurat)  seurat_object = pbmc_small  # tidyseurat  seurat_object %>%  add_count(file) %>%  filter(PC_1 > 0 & n > 40)  # Seurat  pca_emb = Embeddings(object = seurat_object, reduction = "pca")  cell_pca = rownames(pca_emb[pca_emb[,1]>0,])  n =  seurat_object@meta.data %>%  add_count(file) %>%  pull(n)  seurat_object = AddMetaData( object = seurat_object, metadata = n, col.name = 'n')  subset(seurat_object, features = n < 40, cells = cell_pca) |
| --- |

### Supplementary code chunk 2, working code for data import, polishing and exploration

| PBMC_tidy <-  PBMC_integrated %>%    # Clean groups  mutate(Phase = Phase %>% str_remove("^phase_")) %>%    # Extract sample  extract(sample, "sample", "./data/seurat/outs/([a-zA-Z0-9]+)")  # Plot summary statistics  PBMC_tidy %>%  # Data reshaping  pivot_longer(  c(mito.fraction, S.Score, G2M.Score),  names_to="property",  values_to="Value"  ) %>%  # Visualisation  ggplot(aes(sample, Value)) +  geom_boxplot() +  facet_wrap(~property, scales = "free_y" ) |
| --- |

### Supplementary code chunk 3, working code for dimensionality reduction

| pbmc_small_UMAP <-  PBMC_tidy %>%  RunPCA(verbose = FALSE) %>%  RunUMAP(reduction = "pca", dims = 1:15, n.components = 3L)  # 2D plot  pbmc_small_UMAP %>%  ggplot(aes(UMAP_1, UMAP_2, color=seurat_clusters)) +  geom_point(size=0.05, alpha=0.2)  # 3D Plot  pbmc_small_UMAP %>%  plot_ly(  x = ~`UMAP_1`,  y = ~`UMAP_2`,  z = ~`UMAP_3`,  colors = friendly_cols  ) |
| --- |

### Supplementary code chunk 4, working code for clustering

| pbmc_small_cluster <-  pbmc_small_UMAP %>%  FindNeighbors(verbose = FALSE) %>%  FindClusters(method = "igraph")  # Produce summary statistics  pbmc_small_cluster %>%  count(sample, groups, seurat_clusters) |
| --- |

### Supplementary code chunk 5, working code for gene markers identification

| # Identify top 10 markers per cluster  markers <-  pbmc_small_cluster %>%  FindAllMarkers(only.pos = TRUE, min.pct = 0.25, thresh.use = 0.25) %>%  group_by(cluster) %>%  top_n(10, avg_logFC)  # Plot marker genes  pbmc_small_cluster %>%  join_features(features=c("CD3D", "HLA-DRB1")) %>%  ggplot(aes(y=seurat_clusters , x=abundance_SCT, fill=first.labels)) +  geom_density_ridges(bandwidth = 0.2) +  facet_wrap(~feature, nrow = 2) +  coord_flip() |
| --- |

### Supplementary code chunk 6, working code for heatmap construction

| pbmc_small_cluster %>%  sample_n(1000) %>%  join_features(features=markers$gene) %>%  group_by(seurat_clusters) %>%    # Plot heatmap  heatmap(  .row = feature,  .column = cell,  .value = abundance_SCT,  palette_value = circlize::colorRamp2(  c(-1.5, 0, 1.5),  c("purple", "black", "yellow")  )  ) %>%    # Add annotation  add_tile(sample, palette = friendly_cols) %>%  add_point(PC_1) |
| --- |

### Supplementary code chunk 7, working code for cell type inference

| # Join UMAP and cell type info  Pbmc_small_cell_type <-  pbmc_small_cluster %>%  # Data integration  left_join(classification_cluster, by = "seurat_clusters") %>%  left_join(classification_single_cell, by = "cell") %>%  # Reshaping  pivot_longer(  c(seurat_clusters, first.labels_single),  names_to = "classification",  values_to = "value"  ) %>%    # Visualisation  ggplot(aes(  x = classification,  stratum = value,  alluvium = cell,  fill = first.labels, label = value  )) +  scale_x_discrete(expand = c(1, 1)) +  geom_flow() +  geom_stratum(alpha = .5) +  geom_text(stat = "stratum", size = 3) +  coord_flip() |
| --- |

### Supplementary code chunk 8, working code for nested analyses

| pbmc_small_nested <-  pbmc_small_cell_type %>%  # Label lymphoid and myeloid  filter(first.labels != "Platelets") %>%  mutate(cell_class =  if_else(  `first.labels` %in% c("Macrophage", "Monocyte"),  "myeloid",  "lymphoid"  )  ) %>%  # Nesting  nest(data = -cell_class) %>%  # Identification of variable gene features  mutate(variable_genes = map_chr(  data, ~ .x %>%  modelGeneVar() %>%  getTopHVGs(prop=0.05) %>%  FindAllMarkers(only.pos = TRUE, min.pct = 0.25, thresh.use = 0.25) %>%  paste(collapse=", ")  )) |
| --- |

# 
